# Theranostic Liposome–Nanoparticle Hybrids for Drug Delivery and Bioimaging

**DOI:** 10.3390/ijms18071415

**Published:** 2017-07-02

**Authors:** Muharrem Seleci, Didem Ag Seleci, Thomas Scheper, Frank Stahl

**Affiliations:** Institute of Technical Chemistry, Leibniz University of Hanover, Callinstr. 5, 30167 Hanover, Germany; muharremseleci@hotmail.com (M.S.); didemag@hotmail.com (D.A.S.); scheper@iftc.uni-hannover.de (T.S.)

**Keywords:** theranostic liposomes, quantum dots, bioimaging, drug delivery

## Abstract

Advanced theranostic nanomedicine is a multifunctional approach which combines the diagnosis and effective therapy of diseased tissues. Here, we investigated the preparation, characterization and in vitro evaluation of theranostic liposomes. As is known, liposome–quantum dot (L–QD) hybrid vesicles are promising nanoconstructs for cell imaging and liposomal-topotecan (L-TPT) enhances the efficiency of TPT by providing protection against systemic clearance and allowing extended time for it to accumulate in tumors. In the present study, hydrophobic CdSe/ZnS QD and TPT were located in the bilayer membrane and inner core of liposomes, respectively. Dynamic light scattering (DLS), zeta potential (ζ) measurements and fluorescence/absorption spectroscopy were performed to determine the vesicle size, charge and spectroscopic properties of the liposomes. Moreover, drug release was studied under neutral and acidic pH conditions. Fluorescence microscopy and flow cytometry analysis were used to examine the cellular uptake and intracellular distribution of the TPT-loaded L–QD formulation. 3-(4,5-Dimethylthiazol-2-yl)-2,5-diphenyltetrazolium bromide (MTT) assay was utilized to investigate the in vitro cytotoxicity of the formulations on HeLa cells. According to the results, the TPT-loaded L–QD hybrid has adequate physicochemical properties and is a promising multifunctional delivery vehicle which is capable of a simultaneous co-delivery of therapeutic and diagnostic agents.

## 1. Introduction

Nanomedicine is an innovative field with enormous potential for treatment by a combination of smart nanoparticles with small molecules carrying a wide range of functions [1]. Quantum dots (QDs) are one of the promising nanoparticles with excellent fluorescence properties, including broad absorption spectra, narrow emission spectra, high quantum yields, resistance to photobleaching, and high photochemical stability [2,3,4]. Due to these characteristics, they have been explored as fluorescent probes for biomedical applications and are useful in particular for in vivo cell labeling and imaging [5,6,7,8].

Most QDs are typically produced in organic solvents, making them unsuitable for direct use in biomedical and clinical applications. To overcome this limitation, different surface coatings have been applied to increase the hydrophilicity of QDs [9,10]. However, surface modifications often lead to decreases in the QDs’ fluorescence intensity and photostability. In an alternative approach, organic QDs are inserted into lipid bilayers to enhance their hydrophilicity, stability and biocompatibility [11,12,13,14].

Liposomes are self-assembled, spherical lipid-bilayer vesicles that are the most clinically established nanometer-scale systems [15]. They have the capacity to entrap both lipophilic and hydrophobic compounds in a lipid membrane and aqueous core, respectively. Thus, the stability, biocompatibility and solubility of the payloads could be enhanced with the use of several loading strategies.

Liposome–QD (L–QD) hybrid vesicles have shown a great potential for theranostic applications [16]. Tian et al. studied the loading of doxorubicin (DOX) into L–QD hybrid vesicles by the pH-gradient technique; they characterized these DOX-loaded vesicles using dynamic light scattering (DLS) and monitored DOX release [17]. Furthermore, Muthu et al. synthesized folic acid-conjugated theranostic liposomes for the targeted co-delivery of quantum dots and docetaxel [12]. In another study, apomorphine and QDs were integrated into multifunctional liposomes for brain targeting and bioimaging and the results showed that these liposomes can be accumulated to a large extent in the brain [18].

Topotecan (TPT) is a hydrophilic analog of camptothecin. It is a cell-cycle-specific drug and well established for the treatment of several cancers, including ovarian, small-cell lung and cervical cancer [19,20]. However, TPT is unstable in physiological conditions and undergoes a pH-dependent rapid and reversible hydrolysis from a closed lactone ring to the inactive carboxylated form. This causes the loss of the antitumor activity of the drug. To protect TPT from the hydrolysis, liposome has been used for encapsulation [21]. Liposomal encapsulation of TPT enhances its efficacy by protecting it from systemic clearance, allowing greater uptake and extended tissue exposure in solid tumors [22].

In the present study, the aim was to develop multifunctional theranostic liposomes which contain both the model drug TPT and QDs for cell therapy and imaging. For this purpose, L–QD hybrids were synthesized by the incorporation of hydrophobic QD within the lipid bilayer. The biocompatibility of the QDs was increased, retaining its fluorescence characteristics. The pH-gradient technique was used to encapsulate TPT in the aqueous core of the liposome. Thus, TPT therapeutic activity and QD optical properties could be successfully integrated into one nanocarrier.

## 2. Results and Discussion

### 2.1. Synthesis and Characterization of TPT-Loaded Liposomal Formulations

**Figure 1:**
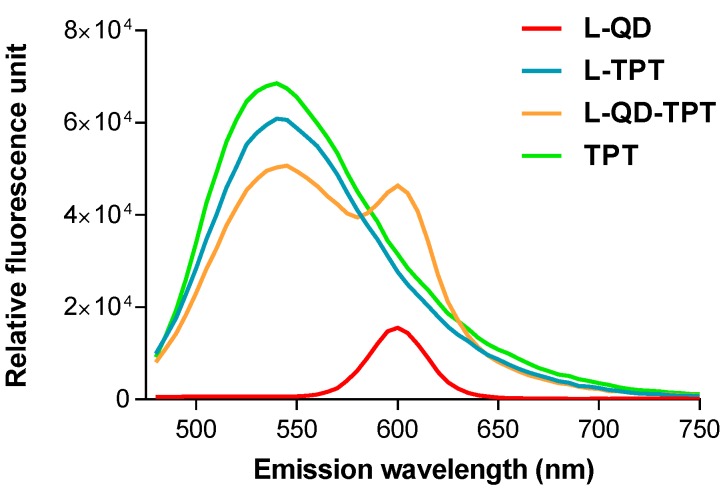
The fluorescence spectra of liposome-quantum dot (L–QD) (red), liposome-topotecan (L-TPT) (turquoise), L–QD–TPT (orange) and free TPT (green). The excitation wavelength was set at 450 nm.

The proportion of the lipids, distearoylphosphatidylcholine (DSPC) and cholesterol, was chosen to be 7:3. DSPC was selected as the bulk phospholipid component instead of egg phosphatidylcholine (EPC) or dimyristoylphosphatidylcholine (DMPC) to increase membrane rigidity, which can also increase drug retention in liposomes [23,24]*.* The influence of cholesterol on the stability of the liposomes has been intensively investigated, which has revealed that cholesterol in sufficient quantity (≤30%) reduces the leakage of loaded materials from liposome by increasing their stability and *decreasing their permeability* [25,26]*.* Furthermore, cholesterol could enhance the hydrophobicity of the membrane [27]*.* Among various synthesis methods, the thin lipid layer hydration method is the most widely-used and simple method for the preparation of the liposomes. pH-gradient technique, which is based on pH gradients as a driving force for the accumulation of the weakly-basic molecules into acidic vesicles, was used to encapsulate TPT into the aqueous core of the liposomes, protecting its active lactone form until released. The fluorescence spectra of the free drug and liposomal formulations were measured and indicated that both QD and TPT peaks could be simultaneously observed in the spectrum (Figure 1). This indicates the coexistence of both molecules in the vesicle. Fluorescence localizations of the molecules are also photographed in a large liposome (Figure S1).

The physicochemical properties of nanoparticles are an important factor in protein interactions [28]. It has been reported that nanoparticles having a size less than 150 nm are more suitable for permeating through the disorganized and leaky microvasculature of the tumor cells. Besides, their more pronounced surface curvature may also reduce the clearance of the particles as a result of reduced interaction with the surface receptors on macrophages [29,30]. In this regard, to determine hydrodynamic diameters and the surface charge of the liposomes, DLS and ζ-potential analysis were carried out (Table 1). The average size of the plain liposomes was measured as ~132 nm. After QD entrapment in the lipid bilayer, a ~6 nm increase in liposome size was detected. This could be associated with the successful encapsulation of the molecules. TPT encapsulation into L–QD did not have an effect on the size (Figure S2). Besides, liposomes were tested for stability upon storage. Over 2 months, no significant differences in size distribution, ζ-potential as well as PDI occurred at 4 °C.

The higher surface charge of nanoparticles affects the amount of protein adsorption as well as protein corona composition on the surface [31,32]. Studies have signified that liposomes which contain highly charged lipids are more susceptible to rapid clearance by the reticuloendothelial system (RES). However, neutral and slightly negatively charged nanoparticles have a longer circulation lifetime and less accumulation in RES [33,34,35]. In the present study, ζ-potential measurements revealed that the surface potential of the liposomes was slightly negative and encapsulations did not show significant alteration in surface charge. TPT encapsulation efficiency was calculated around 40%. QD incorporation into the lipid bilayer of liposomes affected just ~4% of TPT loading through the lipid membrane. Besides, the obtained polydispersity index values were lower than 0.1, confirming the homogeneity of the liposomes (Table 1). Accordingly, the hybrid liposomes have better physicochemical properties.

### 2.2. In Vitro Drug Release

**Figure 2:**
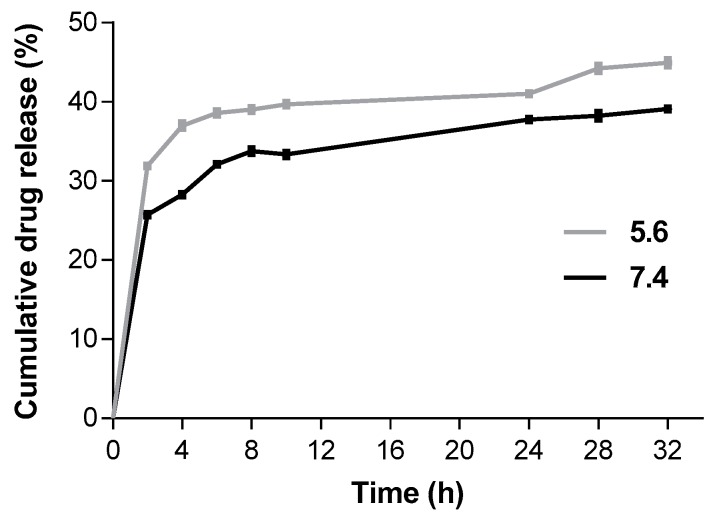
Cumulative drug release profile for L–QD–TPT at mild acidic (pH 5.6) and neutral conditions (pH 7.4). Data are presented as mean ± SD (*n* = 3).

The in vitro drug release profile of L–QD–TPT was investigated by the dialysis method, which is one of the most common methods for the determination of drug release from the nanoparticles. The analysis was carried out in simulated conditions of normal human tissue (pH 7.4) and a tumor microenvironment (pH 5.6) at 37 °C. The acidic environment led to an increased TPT release as compared to neutral conditions (Figure 2). The drug was released with an initial modest burst in the first 4 h followed by slower rates of release up to 32 h. After 32 h, the release rates of TPT were 39% at pH 7.4 and 45% at pH 5.6. The higher release rate at an acidic pH can be ascribed to the solubility of the TPT, which increased with decreasing pH as a result of protonation [36].

### 2.3. Cellular Uptake and Internalization

**Figure 3:**
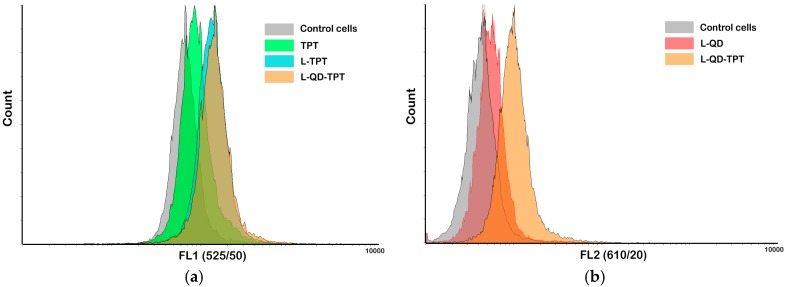
Cellular uptakes of the conjugates were determined in HeLa cells by flow cytometry. TPT, L–TPT, L–QD–TPT (**a**) (excitation at 400 nm, emission filter FL1 (525/50 nm)); L–QD and L–QD–TPT (**b**) (excitation at 400 nm, emission filter FL2 (610/20 nm)).

Cellular uptake of liposomal formulations was examined via flow cytometry. Cells were treated with the samples for 2 h and analyzed using a flow cytometer (BD FACSAria Fusion, Becton, Dickinson and Company, Franklin Lakes, NJ, USA). A UV excitation laser (at 400 nm) was used for all measurements. Higher fluorescence signals (geometric mean values) were obtained from cells treated with the samples compared with untreated control cells. As is clear from Figure 3a, intracellular TPT level for L–TPT (3839 a.u.) and L–QD–TPT (4007 a.u.) in HeLa cells was higher than that of free TPT (2472 a.u.). This difference can be explained by their different uptake mechanisms. Free TPT, a small molecule, is mainly taken into the cells via passive diffusion, whereas liposomes enter the cells through endocytosis [37,38]. Besides, QD-loaded liposomes were also successfully taken up by the cells (Figure 3b). 

**Figure 4:**
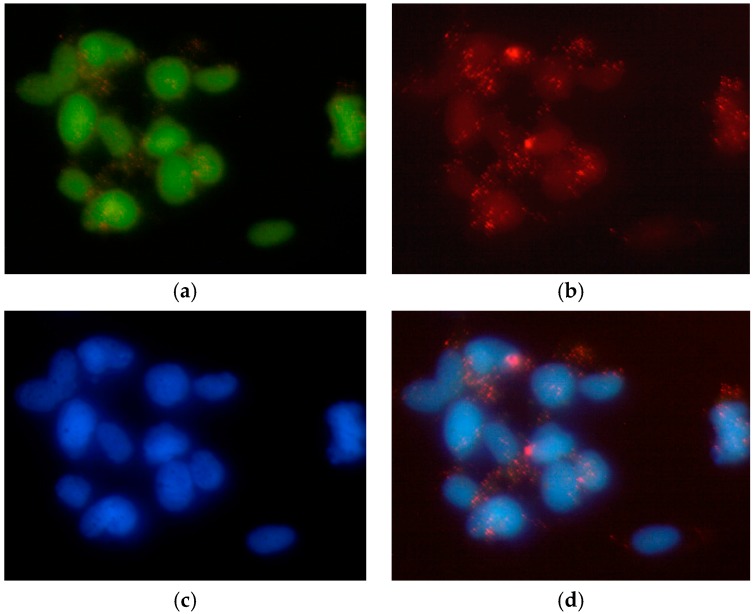
Fluorescence microscopy of HeLa cells after incubation with L–QD–TPT. Liposomes released the payload into the cell (**a**: TPT; **b**: QD). Nuclei were stained with 4′,6-diamidino-2-phenylindole (DAPI) (**c**). The obtained images were merged into the same picture (**d**). All pictures are in 40× magnification.

Fluorescence microscopy was used to observe the cellular internalization of the liposomes. The images were acquired with separate filter sets. In cells treated with L–QD–TPT, the selected fluorescent model drug TPT, which is a topoisomerase-I inhibitor, localized, unsurprisingly, in cell nuclei to exert its toxicity [39,40]**.** The fluorescence from green TPT and blue 4′,6-diamidino-2-phenylindole (DAPI) (nuclear stain) matched well (Figure 4a,c). Besides, the Figure 4b and merged picture (Figure 4d) showed punctate red spots, indicating that QDs had escaped from the endosomes and co-localized in the cytoplasm and partially in the nucleus. The results are compatible with the outcomes of flow cytometry analysis as well as with the literature [12]. Dubertret et al. reported the encapsulation of CdSe/ZnS QDs into phospholipid block-copolymer micelles for in vitro and in vivo imaging. The QD-micelles injected into individual cells of an early embryo and the internalized QDs were localized to both the cytosol and nuclear envelope. Besides, QD cytotoxicity was dose dependent [41].

### 2.4. Cytotoxicity

**Figure 5:**
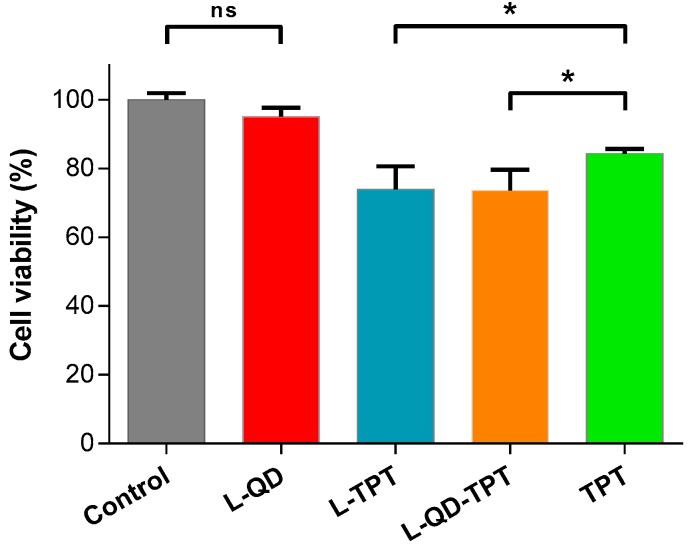
Cytotoxicity of the liposomal formulations and free TPT on HeLa cells. Cells were incubated with, L–QD, L–TPT, L–QD–TPT and free TPT (equivalent concentration of loaded TPT, 2.0 µg/mL) for 24 h. 3-(4,5-Dimethylthiazol-2-yl)-2,5-diphenyltetrazolium bromide (MTT) assay was applied. Error bars represent the standard deviation from the mean (*n* = 3). Data were analyzed using *t*-test, and * *p* < 0.05 was considered significant. ns: not significant.

The cytotoxicity of the liposomal formulations and free TPT on HeLa cells were determined by 3-(4,5-Dimethylthiazol-2-yl)-2,5-diphenyltetrazolium bromide (MTT) assay. Only viable cells with active metabolism are able to convert MTT into purple-colored formazan; the quantity of the formazan is measured by recording the changes in absorbance at 570 nm to 630 nm as the reference wavelength. According to the results, L–QD had no toxic effect due to effective shielding of QD by lipid bilayer from the surroundings. Similar results were also obtained by Chinnathambi and his colleagues. They used phosphoethanolamine(polyethylene glycol)-based phospholipid micelles to encapsulate CdSe/ZnS QDs and QD-micelles showed almost no toxicity in the concentration range of 0–25 µg/mL in HeLa and A549 cell lines exposed for up to 24 h [42], whereas L–TPT and L–QD–TPT showed obvious toxic effects on HeLa cells compared to free TPT for 24 h (*p* < 0.05, Figure 5). This is attributed to the facts that L–TPT and L–QD–TPT were taken up into the cells more efficiently (Figure 3) and that the TPT concentration released from liposomes is somewhat higher compared to that of free TPT. Hao et al. also found similar results regarding the enhancement of the antiproliferation ability of TPT with liposomal encapsulation [43].

## 3. Materials and Methods

### 3.1. Materials

The 3-(4,5-Dimethylthiazol-2-yl)-2,5-diphenyltetrazolium bromide (MTT), 4,6-diamino-2-phenylindol (DAPI), Dulbecco’s modified eagle medium (DMEM) and cholesterol were ordered from Sigma Aldrich (Munich, Germany). CdSe/ZnS core/shell hydrophobic quantum dots (QDs) with an overall diameter of ~5 nm were obtained from PlasmaChem GmbH (Berlin, Germany). Topotecan and 1,2-distearoyl-*sn*-glycero-3-PC (DSPC) were purchased from Cayman Chemical (Ann Arbor, MI, USA).

### 3.2. Synthesis of L–QD Hybrids

L–QD hybrids were synthesized by the thin film hydration method [44]. Briefly, DSPC:Cholesterol (7:3 molar ratio) and 30 µg/mL CdSe/ZnS QD (λ_em_ = 600 nm) solutions in chloroform were added into a round bottom flask. The mixture was placed in an evaporator to obtain a thin QD-containing lipid film and exposed to N_2_ gas for just a few minutes to remove the residual of organic solvents. The dried film was hydrated with 250 mM ammonium sulphate (pH 6.5) at 60 °C, which is above the gel–liquid melting transition temperature (Tm) of all lipids. Small unilamellar vesicles (SUVs) were prepared starting from multilamellar vesicles (MLVs) by using sonication and extruding through 0.4 µm and 0.1 µm pore size polycarbonate membranes in mini-extruder set (Avanti Polar Lipids, Alabaster, AL, USA). Afterward, liposomes were centrifuged at 11,000 rpm for 15 min to remove the excess non-incorporated quantum dots [45].

### 3.3. Encapsulation of Model Drug TPT

L–QD hybrids were loaded actively with TPT using the pH-gradient technique [46]. The obtained lipid-QD dried thin film was hydrated with 250 mM ammonium sulphate (pH 6.5) at 60 °C. After sonication, dialyzed against 10 mM HEPES buffered saline (HBS, pH 7.5) to adjust the exterior pH value of the liposomes as in physiological conditions. The suspension was then mixed with 0.1 mM TPT solution (in 0.9% NaCl) followed by 1 h incubation in a 60 °C water bath. L–QD–TPT was extruded through 0.4 µm and 0.1 µm polycarbonate membranes, respectively. Finally, liposomes were centrifuged at 11,000 rpm for 15 min to remove the excess non-incorporated molecules.

### 3.4. Encapsulation Efficiency

The percent of encapsulation efficiency (EE%) of TPT was calculated according to the following Equation (1):

(1) $$EE{\%}_{\mathrm{TPT}}=\left(W_{\mathrm{en}}/\;W_{\mathrm{total}}\right)\;\times \;100\%$$

where *W*_en_ is the analyzed weight of the drug encapsulated in liposome and *W*_total_ is the initial amount of the drug [47]. It was determined after lysis of the liposomes by diluting purified liposomes in acidic methanol (1% trifluoroacetic acid in methanol). Calibration curves were established with known concentrations of free TPT by fluorescence emission measurements at 530 nm using a fluorospectrometer (NanoDrop 3300, Thermo Fisher Scientific Inc., Waltham, MA, USA).

### 3.5. Characterization

The fluorescence spectra of the free drug and liposomal formulations were measured using a spectrofluorometer. The localizations of QD and TPT in a large liposome were photographed using an Olympus BX41 fluorescence microscope (Shinjuku, Tokyo, Japan) equipped with an Olympus SC30 camera and processed by Image J software [48].

The particle size distribution and zeta potential (ζ) of the liposomes were determined by Zetasizer Nano-ZS (Malvern Instruments, Malvern, UK). The polydispersity index (PDI) was reported as a measure of the width of size distribution. The samples were diluted at the ratio of 1:100 (*v*/*v*) with ddH_2_O and equilibrated for 3 min before the measurements. Measurements were taken three times at room temperature. To test their stability, liposomal formulations were stored at 4 °C in the dark. After 2 months, their size distribution, ζ-potential and PDI were analyzed.

### 3.6. In Vitro Drug Release

The dialysis technique was used for drug release experiments. L–QD–TPT was prepared and transferred into a pre-washed dialysis tubing (Slide-A-Lyzer MINI Dialysis Devices, 10K MWCO, Thermo Fisher Scientific Inc., Waltham, MA, USA). The tubing was immersed in 10 mL of the Phosphate buffered saline (PBS) buffer (pH 5.6 and 7.4), placed in an incubator at 37 °C and stirred at 100 rpm. At specific time intervals, 0.5 mL samples were removed from the release medium and replaced with the same volume of fresh buffer. The amount of released TPT was calculated according to the calibration curves. They were established with a known concentration of free TPT by fluorescence emission measurements at 530 nm using a spectrofluorometer.

### 3.7. Cell Culture

Human cervical cancer cell (HeLa cell) line was provided from the German Collection of Microorganisms and Cell Cultures (DSMZ) (Braunschweig, Germany). Cells were grown in DMEM containing 10% fetal calf serum (FCS) and 1.0% penicillin/streptomycin (P/S). HeLa cells were cultivated in this medium and incubated with samples and reagents at 37 °C in a humidified environment with 5.0% CO_2_.

### 3.8. Cytotoxicity

The MTT (3-(4,5-dimethylthiazol-2-yl)-2,5-diphenyltetrazolium bromide) assays were used to determine the cytotoxicity of the liposomal formulations. Cells (8 × 10^3^) were seeded out in 96-well tissue plates (Sarstedt, Newton, MA, USA) in a volume of 200 µL and cultivated for three days. After this cultivation period, cells were washed once with PBS and treated with L–QD, L–TPT, L–QD–TPT and free TPT for 24 h. The equivalent concentration of free TPT was used in liposomal formulations. The samples were then removed and the cells were incubated in 110 µL/well 10% MTT solution (5.0 mg/mL in PBS) in the medium for 4 h. During this incubation time, formazan complex was produced by the cells. A 100 µL SDS solution (1.0 g SDS in 10 mL 0.01 M HCl) was added to each well to release the purple-colored salt from the cells. After 24 h of incubation, UV-Vis absorption was measured at 570 nm to 630 nm as the reference wavelength using a microplate reader (Epoch BioTek, Winooski, VT, USA).

### 3.9. Cellular Uptake and Internalization

The cellular uptake of TPT and liposomal formulations by HeLa cells was examined through flow cytometry. Cells (5 × 10^5^) were collected and incubated with the samples for 2 h, followed by washing two times with PBS. Just before the analysis, cells were resuspended in 500 µL of PBS and then analyzed in a BD FACSAria Fusion flow cytometer (Becton, Dickinson and Company, Franklin Lakes, NJ, USA). At least 20,000 gated events were observed in total and living cells were gated in a dot plot of forward versus side scatter signals. The dot plot and histogram data were analyzed by Flowing Software 2 [49].

Cellular internalization of L–QD–TPT was determined via fluorescence microscopy studies. HeLa cells were cultivated for 2 days on the chamber slides (8-well µ slides purchased from ibidi GmbH, Munich, Germany) in a volume of 200 µL of the medium. Samples were diluted with the medium and then added to the cells. The cells were incubated for 4 h at 37 °C and washed twice in PBS. Afterwards, 100 µL DAPI solution (1.0 mg/mL) was added to the cells, which were then incubated for 15 min. Cells were washed with PBS once following DAPI staining. Images were taken using an Olympus BX41 fluorescence microscope equipped with an Olympus SC30 camera and processed by Image J software.

### 3.10. Statistical Analysis

Statistical data analysis was performed using the Student’s *t*-test. The difference between two groups was considered to be significant when the *p*-value was less than 0.05.

## 4. Conclusions

The theranostic liposomes, with a mean size of about 135 nm, were developed for the co-delivery of imaging and therapeutic agents. Both hydrophobic QD and hydrophilic TPT were encapsulated into liposomes by thin film hydration and pH-gradient methods, respectively. Thus, bioavailability of a poorly water-soluble molecule was enhanced and moreover, the therapeutic efficacy and stability of the drug were improved. The well-characterized liposomal TPT formulations showed significantly higher cellular uptake as well as higher cytotoxicity than free TPT. QD internalization into cells was achieved and enabled simultaneous imaging. This liposome–nanoparticle hybrid system might offer new opportunities for the development of novel co-delivery platforms.

## Figures and Tables

**Table 1:** Physicochemical properties and EE% of the liposomes.

Samples	Size (nm)	PDI	ζ-Potential (mV)	EE (%)
L	131.8 ± 0.8	0.082	−13.6 ± 1.6	-
L–QD	138.1 ± 0.7	0.014	−7.8 ± 0.1	-
L–TPT	134.1 ± 1.2	0.073	−10.6 ± 0.1	43.8
L–QD–TPT	137.1 ± 1.7	0.069	−6.0 ± 0.1	39.5

The data are presented as the mean ± standard deviation (*n* = 3). PDI: Polydispersity index; ζ-potential: zeta potential; EE: Encapsulation efficiency %.

## Supplementary Materials

Supplementary materials can be found at www.mdpi.com/1422-0067/18/7/1415/s1.
