# Supplementary material for: Theranostic Liposome–Nanoparticle Hybrids for Drug Delivery and Bioimaging
Source: Int J Mol Sci. 2017 Jul 2;18(7):1415. doi: 10.3390/ijms18071415 (PMC5535907; doi:10.3390/ijms18071415)
Supplement: Supplementary file 1 [file ijms-18-01415-s001.pdf]

Electronic Supplementary Information

International Journal of Molecular Sciences

Theranostic liposome-nanoparticle hybrids for drug delivery and bioimaging

Muharrem Seleci, Didem Ag Seleci, Thomas Scheper and Frank Stahl *

Leibniz University of Hanover, Institute of Technical Chemistry, 30167 Hanover, Germany

* Correspondence: stahl@iftc.uni-hannover.de; Tel.: +49 511 762 2968

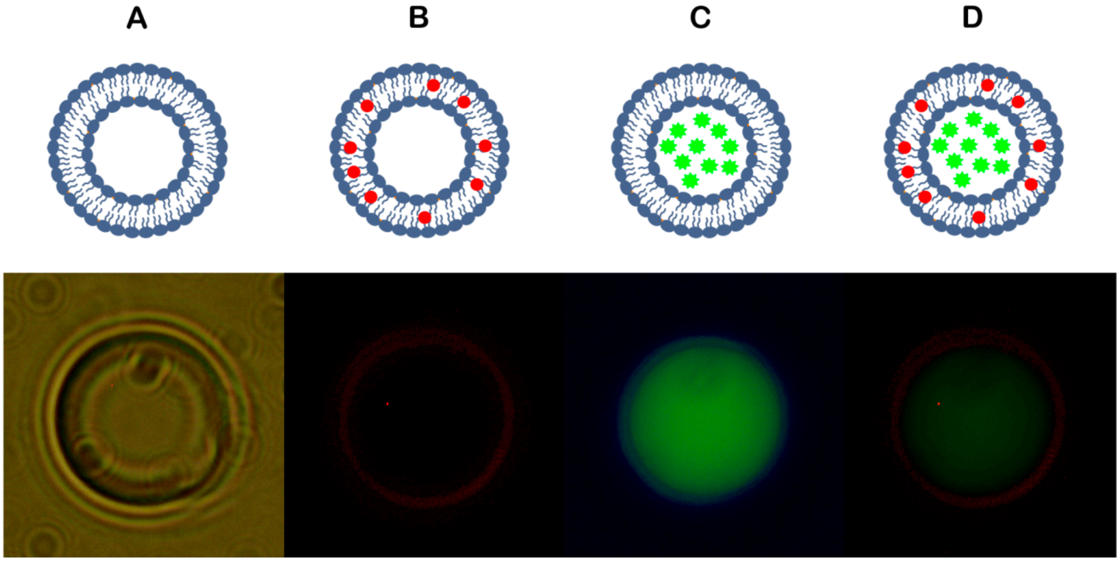

Figure S1. Fluorescence localizations of the molecules in a large liposome, magnification 100×. From left to right: (**A**) L, (**B**) L-QD, (**C**) L-TPT, and (**D**) L-QD-TPT.

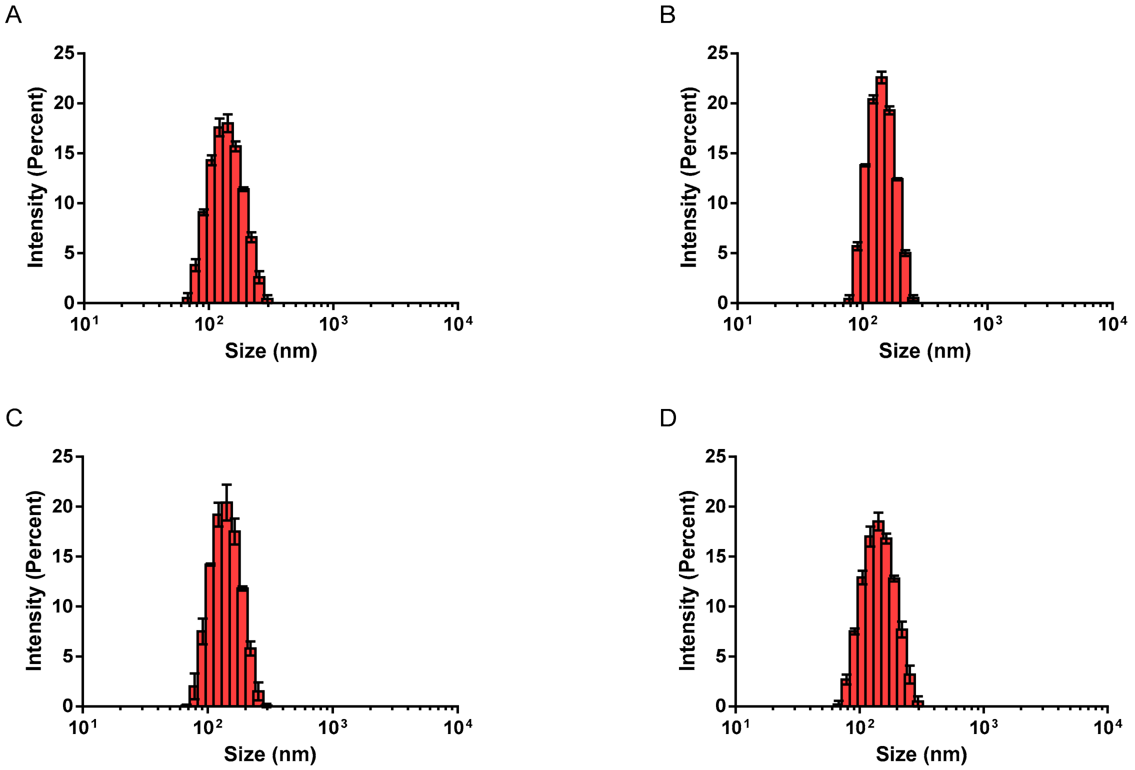

Figure S2. Size distributions of the liposomal formulations: (**A**) L, (**B**) L-QD, (**C**) L-TPT, and (**D**) L-QD-TPT.
